# Peripartum Cardiomyopathy Presenting With Ventricular Tachycardia: A Rare Presentation

**Published:** 2009-05-15

**Authors:** A Puri, R Sethi, B Singh, SK Dwivedi, VS Narain, RK Saran, VK Puri

**Affiliations:** Department of Cardiology, CSM Medical University (Former. King George's Medical University), Lucknow

**Keywords:** Peripartum Cardiomyopathy, Ventricular Tachycardia

## Abstract

A 25-year-old previously asymptomatic pregnant woman at 36 weeks' gestation was noticed to have repetitive monomorphic ventricular tachycardia. A dilated left ventricle with moderately reduced systolic function was found on echocardiographic examination. This is a very rare presentation of peripartum cardiomyopathy (PPCMP) presenting with repetitive monomorphic ventricular tachycardia.

## Clinical Presentation

**Figure 1:**
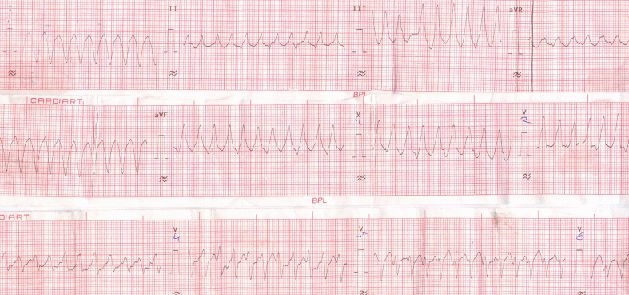
Electrocardiogram at time of admission showing a monomorphic ventricular tachycardia

A 25 years old pregnant woman at 36 weeks gestation presented to the emergency room with the complaints of first episode of acute onset breathlessness and palpitation for 3 days. She did not have any previous history suggestive of heart disease and no family history of any heart disease. She had 2 previous pregnancies and both were uncomplicated. On admission her pulse rate was 200/min regular, blood pressure (BP) was 100/70 mmHg, and chest was clear despite the obvious breathlessness, rest of the physical examination was all with in normal limits. A twelve lead ECG revealed a regular monomorphic ventricular tachycardia (VT) (Figure 1).

**Figure 2:**
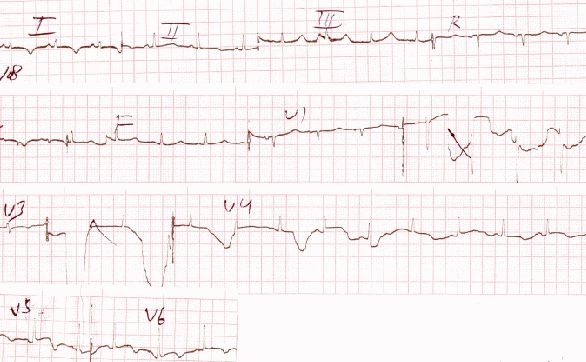
Transient reversion to NSR after intravenous betablockade

**Figure 3:**
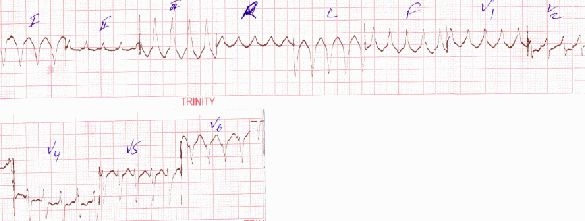
Reappearance of the same monomorphic VT

Immediately after securing an intravenous line a DC cardioversion with 100 J was attempted in the emergency department, the VT transiently reverted to normal sinus rhythm (NSR) but within a few minutes it reappeared and was refractory even after 3 consecutive DC shocks of 360 J. The patient was administered bolus doses of intravenous adenosine (6 mg), followed by intravenous diltiazem (25mg), and finally intravenous amiodarone (150 mg) but there was no response to these drugs. The patient was consistently maintaining a BP of 100/70 mm Hg and was started on amiodarone infusion for 24 hours. Her echocardiogram revealed global hypokinesis with an ejection fraction of 35% with moderate to severe mitral regurgitation and tricuspid regurgitation. A provisional diagnosis of peripartum cardiomyopathy (PPCMP) was kept. The VT persisted at a rate of 200 beats per minute even after 24 hours. The following day the patient was administered intravenous metoprolol 5 mg and she reverted to NSR (Figure 2) which persisted for 12 hours and again the VT reappeared albeit at a slower rate (Figure 3).

**Figure 4:**
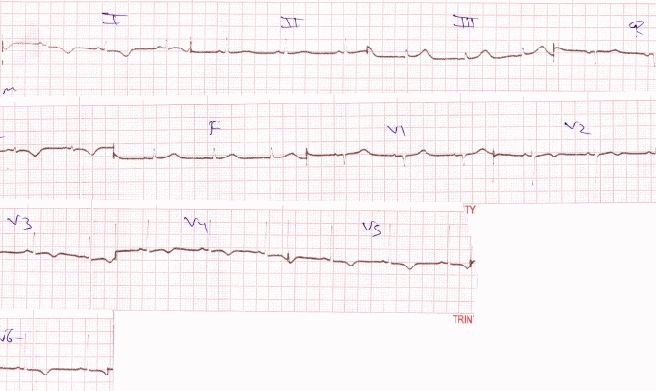
Normal sinus rhythm restored on discharge

After seven days of admission and continuing with a hemodynamically stable VT, she delivered spontaneously a healthy newborn at 37 weeks of pregnancy. In the peripartum period she continued on a hemodynamically stable VT. After the delivery the patient was started on oral amiodarone 1200mg per day and oral metoprolol extended release 100mg per day. Over a period of 3 days, rate of VT decreased gradually and finally reverted to NSR (Figure 4).

The patient was discharged on oral amiodarone which was gradually tapered down to 800 mg per day and oral metoprolol extended release 100mg per day in a stable condition with  a heart rate of 72/min in NSR. She has completed 3 months of follow up and her latest echocardiogram revealed mildly reduced ejection fraction of 50% with trivial mitral regurgitation and no tricuspid regurgitation. 

## Discussion

Peripartum cardiomyopathy (PPCMP) is a rare and some times life-threatening situation of unknown etiology that occurs in previously healthy women. The presentation is most often the development of new onset heart failure. The incidence is approximately 1 per 3,000 to 4,000 live births [1].  PPCMP may some times be unrecognized, due to the non specific nature of the symptoms leading to underestimation of the true incidence. Ventricular tachycardia recognized on electrocardiogram along with a reduction in ejection fraction helped us to reach the diagnosis of PPCMP in our patient, has been reported only once previously [2].Gradual disappearance of her symptoms in the post natal period and normalization of her cardiac function on echocardiogram confirmed our diagnosis. The long-term outcome of PPCMP mainly depends on the recovery of the left ventricular function. Approximately half of the patients recover completely [1,3]. It has been postulated that these patients experience an irregularly distributed focal inflammatory process in the myocardium identifiable as an inflammatory cardiomyopathy or a myocarditis [4] and eventually leading to left ventricular dysfunction. Interestingly the patient was transiently reverted to NSR with metoprolol and the effect of beta-blockers on circulating levels of inflammatory and anti-inflammatory cytokines in patients with dilated cardiomyopathy has been described [5]. Patients whose left ventricular dysfunction does not resolve within six months following delivery are known to have an extremely high mortality rate [1,3,6]. There may also be a genetic predisposition to the development of PPCMP [7]. Hence, the ventricular arrhythmia in the present case was expected to resolve as left ventricular function recovered and the patient was continued with beta blockers and amiodarone. To the best of our knowledge, a case of PPCMP presenting with repetitive monomorphic ventricular tachycardia is a very rare presentation of this disease.
